# Supplementary material for: Just 2% of SARS-CoV-2-positive individuals carry 90% of the virus circulating in communities
Source: medRxiv. 2021 Mar 5:2021.03.01.21252250. Preprint. [Version 1] doi: 10.1101/2021.03.01.21252250 (PMC7941634; doi:10.1101/2021.03.01.21252250)
Supplement: 1 [file NIHPP2021.03.01.21252250-supplement-1.pdf]

medRxiv preprint doi: https://doi.org/10.1101/2021.03.01.21252250; this version posted March 5, 2021. The copyright holder for this preprint (which was not certified by peer review) is the author/funder, who has granted medRxiv a license to display the preprint in perpetuity.
It is made available under a CC-BY 4.0 International license .

SUPPLEMENTAL INFORMATION

Supplementary Table S1. Studies from which viral loads in symptomatic individuals were derived*

STUDY	HOSPITAL, COUNTRY	GENDER (MALE/FEMALE)	AGE	METHOD
**TO ET AL.[51]**	Princess Margaret Hospital and Queen Mary Hospital, Hong Kong	13/10	37-75	Posterior oropharyngeal saliva followed by RT-qPCR
**TO ET AL.[52]**	Princess Margaret Hospital and Queen Mary Hospital, Hong Kong	7/5	37-75	Posterior oropharyngeal saliva followed by RT-qPCR
**ZHANG ET AL.[53]**	Wuhan Pulmonary Hospital, China	NA	NA	Saliva from oral swab followed by RT-qPCR
**HANEGE ET AL.[54]**	Goztepe Education and Research Hospital, Turkey	11/18	26-70	Self-collected saliva followed by RT-qPCR
**PROCOP ET AL.[55]**	Cleveland Clinic, USA	25/14	18-82	Self-collected saliva followed by RT-qPCR
**ZHENG ET AL.[56]**	First Affiliated Hospital, College of Medicine, China	58/38	44-64	Self-collected saliva after deep cough followed by RT-qPCR
**YOON ET AL.[57]**	Korea University Guro Hospital, Korea	0/2	46-65	Self-collected saliva followed by RT-qPCR
**WYLLIE ET AL.[18]**	Yale New Haven Hospital, USA	NA	NA	Self-collected saliva followed by RT-qPCR
**YOKOTA ET AL.[58]**	Hokkaido University Hospital, Japan	25/17	27-93	Self-collected saliva followed by RT-qPCR
**ZHU ET AL.[59]**	Central Hospital of Xiangtan, China	16/16	34-54	Self-collected saliva followed by RT-qPCR

* All studies indicated that saliva samples were self-collected from COVID-19 patients at the indicated locations. In all cases, the authors reported virus concentrations in the original saliva sample.

medRxiv preprint doi: https://doi.org/10.1101/2021.03.01.21252250; this version posted March 5, 2021. The copyright holder for this preprint (which was not certified by peer review) is the author/funder, who has granted medRxiv a license to display the preprint in perpetuity.
It is made available under a CC-BY 4.0 International license .

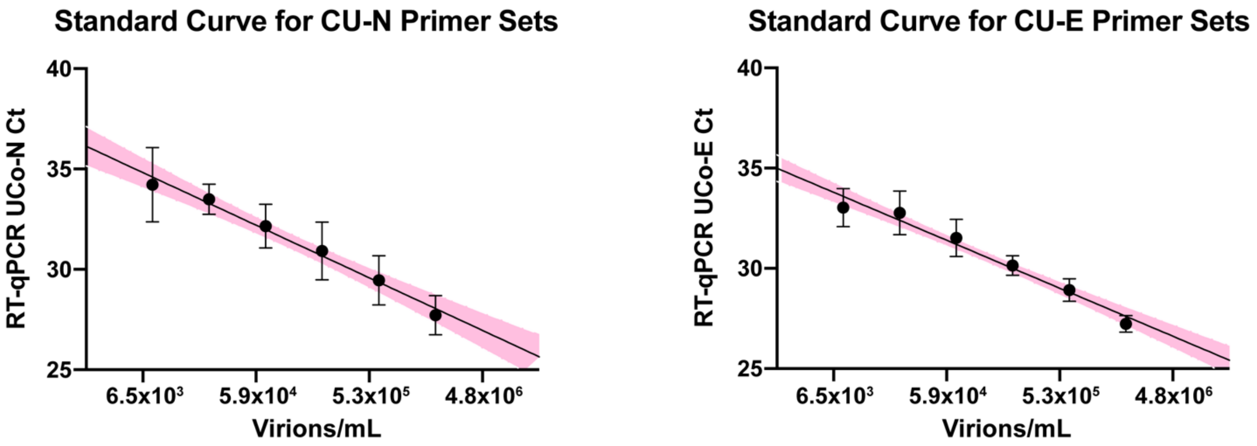

Figure S1. **Standard curves for primer sets used in this study.** 10,000 virions/µL of heat deactivated SARS-CoV-2 virus was spiked into negative saliva specimens from 6 different individuals and incubated for 30 minutes at 95ºC. Samples were diluted to indicated concentrations using heat-treated saliva without SARS-CoV-2 addition from the same individuals. The standard curve generated from the linear regression analysis is illustrated with 95% confidence interval.

medRxiv preprint doi: https://doi.org/10.1101/2021.03.01.21252250; this version posted March 5, 2021. The copyright holder for this preprint (which was not certified by peer review) is the author/funder, who has granted medRxiv a license to display the preprint in perpetuity.
It is made available under a CC-BY 4.0 International license .

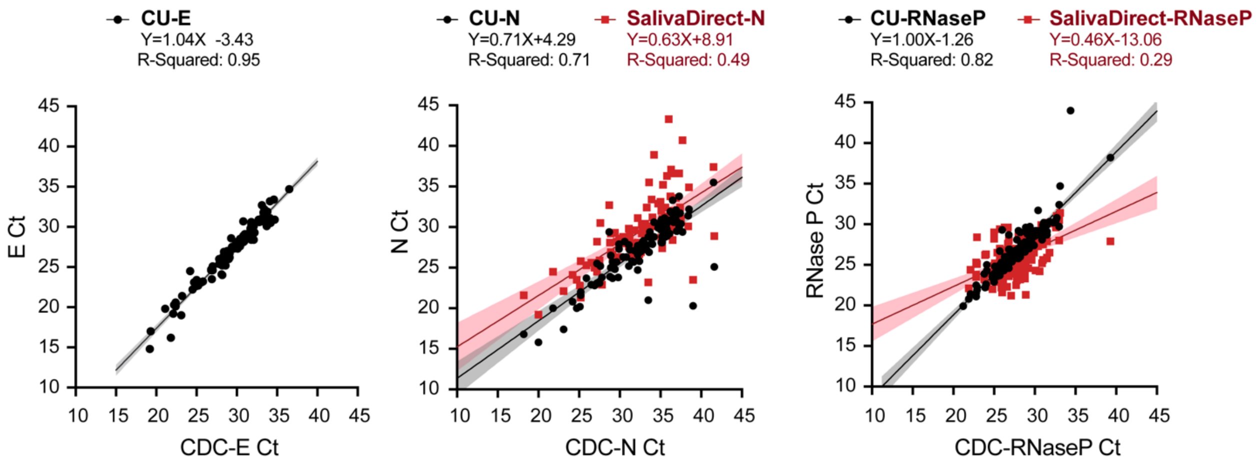

Figure S2. Correlation of Ct values between different primer sets used to quantify saliva viral load. Using 105 SARS-CoV-2 positive saliva samples, we examined the Ct values obtained with different RT-qPCR multiplex assays and compared them via correlation analysis. For 105 virus-positive saliva samples, 8 different Ct values were generated all in one day from each sample, in a side-by-side direct analysis of the performance of each primer set. Ct values from the Centers for Disease Control primers (CDC-E, CDC-N or CDC-RNaseP) are reported on the X-axes. On the Y-axes are plotted the corresponding Ct values resulted from our university screening primers (CU-E, CU-N or CU-RNaseP) and primer sets used in the SalivaDirect [20] test (SalivaDirect-N and SalivaDirect-RNase P) primer sets.
